# Risk Factors for Intrapartum Anorectal Mucosal Lacerations and Rectovaginal Fistula: A Retrospective Comparative Study

**DOI:** 10.31662/jmaj.2023-0131

**Published:** 2024-02-27

**Authors:** Shunji Suzuki

**Affiliations:** 1 Department of Obstetrics and Gynecology, Japanese Red Cross Katsushika Maternity Hospital, Tokyo, Japan

**Keywords:** vaginal delivery, anorectal mucosal lacerations, fourth-degree perineal lacerations, rectovaginal fistula, risk factors, episiotomy

## Abstract

**Introduction:**

We examined the risk factors for fourth-degree perineal lacerations (intrapartum anorectal mucosal lacerations) and rectovaginal fistulas as one of the later complications.

**Methods:**

We reviewed the obstetric records of all singleton vaginal deliveries after 22 weeks of gestation at our institute between January 2006 and December 2018 (n = 19,370).

**Results:**

Of the 19,370 deliveries, 61 had fourth-degree perineal lacerations (0.31%). Of the 61 women, 5 (8.2%) developed rectovaginal fistulas 2-3 weeks after their deliveries. Upon multivariate analysis, nulliparity (Adjusted odds ratios (OR) 3.58, 95% confidence interval (CI) 1.6-8.1, p < 0.01), midline episiotomy (Adjusted OR 2.10, 95% CI 1.0-4.2, p = 0.03), vacuum extraction (Adjusted OR 7.01, 95% CI 3.5-14, p < 0.01), and forceps delivery (Adjusted OR 22.0, 95% CI 7.8-61, p < 0.01) were independently associated with fourth-degree perineal lacerations, while mediolateral episiotomy (Adjusted OR infinity, 95% CI 2.1-infinity, p = 0.03) and forceps delivery (Adjusted OR infinity, 95% CI 14.5-infinity, p = 0.01) were independently associated with rectovaginal fistulas. In addition, in the women with fourth-degree perineal lacerations, mediolateral episiotomy was associated with rectovaginal fistulas (OR infinity, 95% CI 1.8-infinity, p = 0.04).

**Conclusions:**

Midline episiotomy and instrument-assisted delivery are independent risk factors for fourth-degree perineal lacerations after vaginal delivery. Mediolateral episiotomy and forceps delivery were independently associated with rectovaginal fistulas. Once fourth-degree perineal lacerations occurred, women with mediolateral episiotomies were more likely to develop rectovaginal fistulas.

## Introduction

Fourth-degree perineal lacerations are lacerations of the perineal body, entire anal sphincter complex, and anorectal mucosa (intrapartum anorectal mucosal lacerations). It is an uncommon, unpredictable injury that obstetricians may face ^(1)^. Effective repair of the lacerations will require a deep knowledge of perineal anatomy and surgical technique ^(2)^. Women with fourth-degree perineal lacerations have been observed to have bowel symptoms and demonstrate persistent combined defects of the internal and external anal sphincter ^(3)^. Rectovaginal fistulas are one of the most serious later complications of the lacerations. The risk factors for fourth-degree perineal lacerations have been reported to include nulliparity, midline episiotomy, persistent occiput posterior position of the fetus during labor, large fetal birth weight, and operative vaginal delivery ^(4), (5)^; however, those of rectovaginal fistulas has not been well investigated.

In this study, we examined (1) the risk factors for fourth-degree perineal lacerations and (2) rectovaginal fistulas in women after delivery and those with fourth-degree perineal lacerations.

## Materials and Methods

The protocol for this study was approved by the Ethics Committee of the Japanese Red Cross Katsushika Maternity Hospital (K2018-28). Informed consent concerning the retrospective analysis was obtained from each subject during their hospital visit.

To perform the retrospective comparative analyses, we reviewed the obstetric records of all singleton vaginal deliveries after 22 weeks of gestation at our institute, excluding cases of intrauterine fetal demise between January 2006 and December 2018 (n = 19,370). Of these, 61 had fourth-degree perineal lacerations (0.31%), and 5 of them (8.2%) developed rectovaginal fistulas. No women who experienced rectovaginal fistulas had perineal lacerations lower than the fourth degree.

To examine the risk factors for fourth-degree perineal lacerations, the control group was defined as the rest of the 19,309 deliveries (99.6%) without fourth-degree perineal lacerations. Of these, 236 (1.2%) had third-degree perineal lacerations, and 19,073 (98.5%) had perineal lacerations lower than the second-degree.

In our institute, the rectal mucosa is repaired using a 4-0 or 3-0 absorbable suture with a noncutting needle ^(6), (7)^. The first layer of the mucosal repair is performed with a submucosal single intermittent ligature suture spaced approximately 2 mm apart and stopped just at the anal margin limit, after which, the knot is tied. The second layer of sutures is performed to protect the mucosal suture layer. A separate repair of the internal and external anal sphincters is performed to prevent incontinence risk from postpartum internal anal sphincter defects. Postoperatively, a woman with a fourth-degree laceration is managed as an inpatient with an intravenous administration of Cephem antibiotics for 3 days, and vaginal cleansing is performed every day until the first bowel movement. After the first bowel movement, she will be discharged from the hospital after a rectal examination to confirm that there are no problems in her rectal mucosa. Several weeks of therapy with a stool softener is also given to minimize the potential for repair breakdown from straining during defecation.

To examine the risk factors for rectovaginal fistulas, in this study, 19,365 women without a rectovaginal fistula were defined as the first control group to examine the risk factors for the injury. In addition, 56 women (93.4%) without a rectovaginal fistula following fourth-degree perineal lacerations were defined as the second control group to examine the risk factors for developing a rectovaginal fistula in women with fourth-degree perineal lacerations.

Maternal and fetal factors such as maternal age, parity, neonatal birth weight, oxytocin use (either for induction or augmentation), type of episiotomy (midline, mediolateral, or no episiotomy), and method of delivery (normal, vacuum, forceps, or vaginal breech delivery) were extracted from the obstetric records.

Data are presented as percentages (%). The statistical software SAS version 8.02 (SAS Institute, Cary, NC, USA) was used for statistical analyses. To assess the association between the presence of fourth-degree perineal lacerations and rectovaginal fistula and each set of discrete variables, a chi-square analysis with Yates’ continuity correction was performed. Logistic regression analysis was used to identify independent risk factors associated with fourth-degree perineal lacerations and rectovaginal fistulas. The level of statistical significance used was a probability value of less than 0.05. Variables used in the multivariate model were those that had shown a significant association with the occurrence of fourth-degree perineal lacerations and rectovaginal fistulas on univariate analysis. Odds ratios (ORs) and 95% confidence intervals (CIs) were also calculated.

## Results

### (1) Risk factors for fourth-degree perineal lacerations

Table 1 shows the clinical characteristics and univariate association with fourth-degree perineal lacerations. As shown, nulliparity (crude OR 6.89, 95% CI 3.2-15, p < 0.01), oxytocin use (crude OR 1.98, 95% CI 1.2-3.3, p = 0.01), midline episiotomy (crude OR 3.32, 95% CI 1.8-6.0, p < 0.01), mediolateral episiotomy (crude OR 2.38, 95% CI 1.3-4.4, p < 0.01), vacuum extraction (crude OR 14.4, 95% CI 8.3-25, p < 0.01), forceps delivery (crude OR 44.6, 95% CI 20-99, p < 0.01), and breech delivery (crude OR 10.4, 95% CI 2.7-40, p < 0.01) were associated with fourth-degree perineal lacerations. Upon multivariate analysis, nulliparity (adjusted OR 3.58, 95% CI 1.6-8.1, p < 0.01), midline episiotomy (adjusted OR 2.10, 95% CI 1.0-4.2, p = 0.03), vacuum extraction (adjusted OR 7.01, 95% CI 3.5-14, p < 0.01), and forceps delivery (adjusted OR 22.0, 95% CI 7.8-61, p < 0.01) were independently associated with fourth-degree perineal lacerations.

**Table 1.** Clinical Characteristics and Univariate Association with and without Fourth-Degree Perineal Lacerations.

Fourth-degree lacerations	No			Yes	*P-value
	≤Second-degree lacerations	Third-degree lacerations	Total		
	19,073	236	19,309	61	
Maternal age ≥40 years	807 (4.2)	7 (3.0)	814 (4.2)	3 (4.9)	0.79
Nulliparity	10,020 (52.5)	180 (76.3)	10,200 (52.8)	54 (88.5)	<0.01
Neonatal birth weight ≥3500g	2,141 (11.2)	18 (7.6)	2,159 (11.2)	11 (18.0)	0.09
Oxytocin use					
None	14,847 (77.8)	182 (77.2)	15,029 (77.8)	39 (63.9)	-
Yes	4,226 (22.2)	54 (22.8)	4,280 (22.2)	22 (36.1)	0.01
Episiotomy					
None	11,705 (61.4)	126 (53.4)	11,831 (61.3)	22 (36.1)	-
Midline episiotomy	3,367 (17.6)	40 (16.9)	3407 (17.6)	21 (34.4)	<0.01
Mediolateral episiotomy	4,001 (21.0)	70 (29.7)	4071 (21.0)	18 (29.5)	<0.01
Delivery mode					
Normal delivery	17,450 (91.4)	204 (86.4)	17,654 (91.4)	24 (39.3)	-
Vacuum extraction	1,356 (7.1)	26 (11.0)	1382 (7.2)	27 (44.3)	<0.01
Forceps delivery	126 (0.7)	6 (2.5)	132 (0.7)	8 (13.1)	<0.01
Breech delivery	141 (0.7)	0 (0)	141 (0.7)	2 (3.3)	<0.01

Data are presented as percentages (%) *P-value vs. total women without fourth-degree perineal lacerations.

### (2) Risk factors for rectovaginal fistulas in all women after delivery and those following fourth-degree perineal lacerations

In this study, of the 61 women with fourth-degree perineal lacerations, 5 (8.2%) developed rectovaginal fistulas 2-3 weeks after their deliveries. Table 2 shows the clinical characteristics and univariate association with rectovaginal fistulas in all populations in this study. Mediolateral episiotomy (crude OR infinity, 95% CI 3.8-infinity, p < 0.01) and instrument-assisted delivery (vacuum extraction: crude OR infinity, 95% CI 9.8-infinity, p < 0.01, forceps delivery: crude OR infinity, 95% CI 65-infinity, p < 0.01) were associated with rectovaginal fistulas. Upon multivariate analysis, mediolateral episiotomy (adjusted OR infinity, 95% CI 2.1-infinity, p = 0.03) and forceps delivery (adjusted OR infinity, 95% CI 14.5-infinity, p = 0.01) were independently associated with rectovaginal fistulas. Table 3 shows the clinical characteristics and univariate association with rectovaginal fistulas in women with fourth-degree perineal lacerations only. In addition, mediolateral episiotomy was associated with rectovaginal fistulas (OR infinity, 95% CI 1.8-infinity, p = 0.04).

**Table 2.** Clinical Characteristics and Univariate Association with Rectovaginal Fistulas.

Rectovaginal fistula	No	Yes	P-value
	19,365	5	
Maternal age ≥40 years	817 (4.2)	0	1
Nulliparity	10,250 (52.9)	5 (100)	0.097
Neonatal birth weight ≥ 3500g	2,169 (11.2)	1 (20)	1
Oxytocin use			
None	15,063 (77.8)	5 (100)	-
Yes	4,302 (22.2)	0	0.51
Episiotomy			
None	11,852 (61.2)	0	-
Midline episiotomy	3,428 (17.8)	0	1
Mediolateral episiotomy	4,085 (21.1)	5 (100)	<0.01
Delivery mode			
Normal delivery	17,678 (91.2)	0	-
Vacuum extraction	1,406 (7.2)	3 (80)	<0.01
Forceps delivery	140 (0.7)	2 (20)	<0.01
Breech delivery	143 (0.7)	0	1

Data are presented as percentages (%).

**Table 3.** Clinical Characteristics and Univariate Association with Rectovaginal Fistulas in Women with Fourth-Degree Perineal Lacerations.

Rectovaginal fistula	No	Yes	P-value
	56	5	
Maternal age ≥40 years	3 (5.4)	0	1
Nulliparity	50 (89)	5 (100)	1
Neonatal birth weight ≥3500g	10 (18)	1 (20)	1
Oxytocin use			
None	34 (61)	5 (100)	-
Yes	22 (39)	0	0.21
Episiotomy			
None	21 (38)	0	-
Midline episiotomy	21 (38)	0	1
Mediolateral episiotomy	14 (25)	5 (100)	0.04
Delivery mode			
Normal delivery	23 (41)	0	-
Vacuum extraction	24 (43)	3 (80)	0.29
Forceps delivery	8 (14)	2 (20)	0.16
Breech delivery	2 (3.6)	0	1

Data are presented as percentages (%).

Of the 5 cases with rectovaginal fistulas, 3 were successfully resolved spontaneously; however, 2 required fistula closure.

## Discussion

### (1) Risk factors for fourth-degree perineal lacerations

This study showed that nulliparity, midline episiotomy, and instrument-assisted cephalic delivery are independent risk factors for fourth-degree perineal lacerations after vaginal delivery. These tendencies support some previous observations concerning the risk factors for severe perineal lacerations ^(4), (5), (8), (9)^. Concerning severe perineal lacerations during vaginal delivery, there may be multiple obstetric contributory factors despite routine episiotomy, among them, nulliparity and instrument-assisted vaginal delivery. In an earlier study in Japan ^(8)^, a shorter attendant experience was an additional risk factor for severe perineal lacerations. We did not conduct the examination concerning attendant experience because our institute has only employed obstetricians who have completed initial training at a specialized perinatal facility. Therefore, the instrument-assisted vaginal delivery was only recommended to be performed after careful evaluation ^(8), (9)^.

### (2) Risk factors for rectovaginal fistulas in all women after delivery and those following fourth-degree perineal lacerations

In this study, no women who experienced rectovaginal fistulas had perineal lacerations lower than the fourth degree. Of the 61 with fourth-degree perineal lacerations, 5 (8.2%) developed rectovaginal fistulas. Notably, mediolateral episiotomy and forceps delivery were independently associated with rectovaginal fistulas. In addition, as a new finding, it was observed that once fourth-degree perineal lacerations occurred, women who had mediolateral episiotomy were more likely to develop a rectovaginal fistula. The small sample size of the current study may be one of the major limitations; however, a rectovaginal fistula is a serious complication. Therefore, the findings cannot be overlooked.

The results are understandable because forceps delivery will place a greater burden on the perineum than vacuum extraction ^(10)^. Furthermore, to avoid serious lacerations, it is necessary to perform mediolateral episiotomy rather than midline episiotomy. In addition, rectovaginal fistulas developed because of an incomplete repair of postpartum fourth-degree perineal lacerations. Rectovaginal fistula repair after failed primary repair may be uncommon. During the study period, Cephem antibiotics were administered intravenously, and vaginal irrigation was performed daily in all women with fourth-degree lacerations; however, this was not effective in 5 cases who developed a rectovaginal fistula. Eventually, 3 cases were spontaneously repaired, and 2 cases were cured by repair surgery. For the repair of anorectal mucosal lacerations, a combination of a horizontal mattress suture and running suture has been reported to be useful without complications ^(6), (11)^. Although the suture method may be amenable to symmetrical anorectal lacerations following midline episiotomy, irregular lacerations that are unevenly stretched after mediolateral episiotomy cannot be sutured typically. The situation may also be related to the cause of the failed primary repair. If the sutures are not evenly pressurized, vulnerable areas will occur at the sutures, and if the blood flow is not sufficient, prolonged ischemia, infection, and necrosis will be more likely to occur ^(6), (11), (12)^. Uneven pressure from fecal masses can also lead to the formation of a fistula in the vulnerable areas of the sutures.

We understand that there are various limitations other than the small sample size in the current study. We did not examine the rotation of the fetal head, indications for instrumental delivery, and the skill of the physicians ^(4), (5), (8), (9)^. In particular, the differences in suturing techniques between our physicians might be very important ^(6), (8)^. In addition, since rectal examinations were not performed for all subjects 2-3 months after the delivery, it cannot be denied that there are women who have rectovaginal fistulas but do not have subjective symptoms.

### Conclusions

Midline episiotomy and instrument-assisted delivery are independent risk factors for fourth-degree perineal lacerations after vaginal delivery. Mediolateral episiotomy and forceps delivery were independently associated with rectovaginal fistulas. Once fourth-degree perineal lacerations occurred, women with mediolateral episiotomies were more likely to develop rectovaginal fistulas.

## Article Information

### Conflicts of Interest

None

### Author Contributions

Shunji Suzuki: project development, data management, data analysis, and manuscript writing and editing.

### Approval by Institutional Review Board (IRB)

The study protocol was approved by the Ethics Committee of the Japanese Red Cross Katsushika Maternity Hospital (K2018-28).

### Imformed Consent

Patients’ informed consent for the publication of this report was obtained.
